# Supplementary material for: A Global Screen for Assembly State Changes of the Mitotic Proteome by SEC-SWATH-MS
Source: Cell Syst. 2020 Feb 26;10(2):133–155.e6. doi: 10.1016/j.cels.2020.01.001 (PMC7042714; doi:10.1016/j.cels.2020.01.001)

Q9UMS4 | PRP19_HUMAN | PRPF19 NMP200 PRP19 SNEV
Monomer MW [kDa]: 55.181 Monomer expected elution fraction: 45

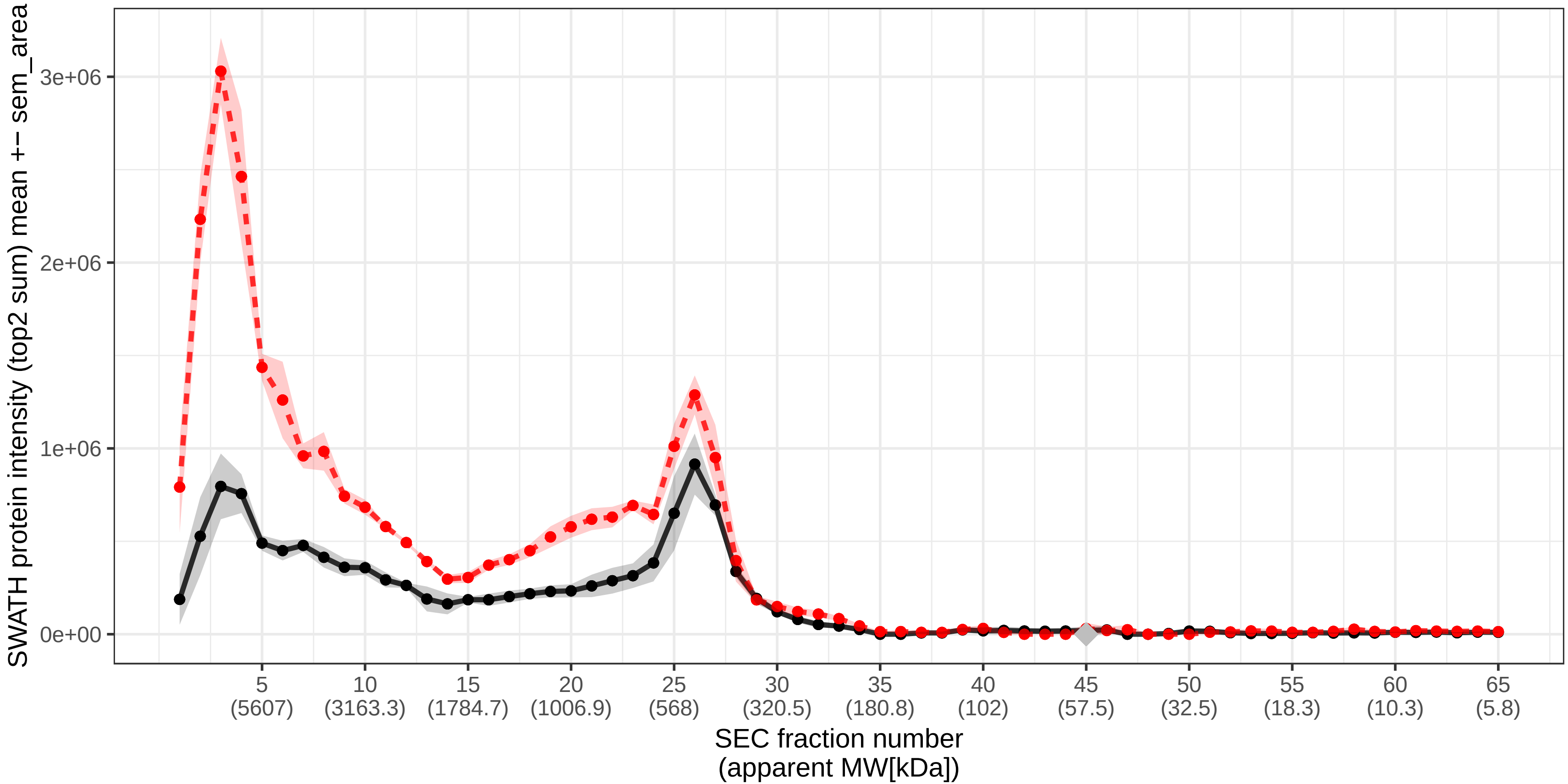

Supplement: Data S1. SEC-SWATH-MS Protein Chromatograms, Related to Figure 1 [file mmc6.zip › SECchrom_Q9UMS4_PRP19_HUMAN_PRPF19_NMP200_PRP19_SNEV.pdf]
